# Two-Way Transmission for Low-Latency and High-Reliability 5G Cellular V2X Communications

**DOI:** 10.3390/s20020386

**Published:** 2020-01-10

**Authors:** Dinh-Thuan Do, Tu-Trinh Thi Nguyen, Chi-Bao Le, Jeong Woo Lee

**Affiliations:** 1 Wireless Communications Research Group, Faculty of Electrical and Electronics Engineering, Ton Duc Thang University, Ho Chi Minh City 700000, Vietnam; dodinhthuan@tdtu.edu.vn; 2 Faculty of Electronics Technology, Industrial University of Ho Chi Minh City (IUH), Ho Chi Minh City 700000, Vietnam; nguyenthitutrinh@iuh.edu.vn (T.-T.T.N.); lechibao@iuh.edu.vn (C.-B.L.); 3 School of Electrical and Electronics Engineering, Chung-Ang University, Seoul 06974, Korea

**Keywords:** V2X network, NOMA, outage probability

## Abstract

As one of key technologies of future networks, vehicle-to-everything (V2X) communication has recently been proposed to improve conventional vehicle systems in terms of traffic and communications. Main benefits of using V2X are efficient and safe traffic as well as low-latency communications and reliable massive connections. Non-orthogonal multiple access (NOMA) scheme was introduced as a promising solution in the fifth-generation (5G) mobile communications, by which quality-of-service (QoS) requirements of many 5G-enabled applications are satisfied as a result of improved network throughput and lower accessing and transmission latency. In this paper, we study NOMA-based communications between vehicles equipped with multiple antennas over Nakagami-m fading channels in V2X networks, in which uplink and downlink transmission between two vehicles with upper controller are supported by a road side unit (RSU) to increase the capacity rather than simply be connected to the base station. In the NOMA-V2X system under study, the outage probability depends on the power allocation factor of RSU transmission and the operation of successive interference cancellation (SIC) at vehicles. Analyses and simulations verify that the outage performance of NOMA-V2X system are mainly affected by fading parameters, levels of imperfect SIC, and power allocation factors.

## 1. Introduction

Vehicle-to-everything (V2X) networks are expected to enable various applications of emerging and promising technologies in Internet of Things (IoT). V2X can provide a safer and more efficient driving experience for our future daily life, and introduce services related to drivers, passengers, pedestrians, vehicles, and traffic. V2X communication will play a vital role in safety-critical and delay-sensitive services [1,2] because it can provide low latency and high reliability. Recently, the third generation partnership project (3GPP) provided Long Term Evolution(LTE)-based cellular V2X solutions for public safety services by extending the 3GPP LTE device-to-device (D2D) communication technology. In a dense environment causing severe data congestion, however, the LTE-based vehicle networks may meet new challenges to satisfy requirements of low latency, high reliability, and a large number of connected devices [3,4,5]. These requirements are different from those of LTE D2D communications in which easier demands are required by V2X applications. The dense networks cannot avoid severe data congestion and low access efficiency because the limited spectrum range have not been fully and efficiently implemented in the existing LTE networks by employing the orthogonal multiple access (OMA) scheme [6]. Recently, the non-orthogonal multiple access (NOMA) scheme was proposed as an efficient radio access technology to overcome aforementioned drawback of OMA and fully utilize the capacity area, and is considered as a potential solution for fifth-generation (5G) wireless networks [7]. In NOMA, transmitters send multiple signals in a superimposed form by exploiting power domain multiplexing, and receivers facilitate successive interference cancellation (SIC) to detect desired signals [8]. NOMA is able to provide lower system delays, higher reliability, higher transmission rates, and lower-cost service requirements in comparison with the OMA scheme [9,10].

Recently, cooperative communications have been actively studied, whose major benefits are wider cell coverage and higher spectrum efficiency (SE). Cooperative power domain non-orthogonal multiple access (CNOMA) have received a lot of attention as an example of cooperative communications [11]. In CNOMA, near users (NU) obtain their own signals by decoding the superposed signal and forward far users’ (FU) signals to FUs who have weaker links with a base station (BS). CNOMA shows performance improvement in terms of outage probability and average sum rate. To maximize fairness for paired user group and further improve the performance of CNOMA, power allocation among multiple users are considered under a half-duplex mode [12]. To obtain higher SE, a full-duplex mechanism has recently been proposed for CNOMA [13], in which downlink (DL) full-duplex CNOMA system is studied in terms of outage probability and ergodic rate without direct link between BS and FU. Power allocation architecture was also presented for the purpose of maximizing the minimum achievable rate and minimizing outage probability of users.

Most of related works have focused on DL transmission, in which NUs support FUs’ transmissions while uplink (UL) transmission is also important for cooperative relaying. Xiao et al. designed time and power allocations for two-hop decode-and-forward (DF) NOMA relaying protocol to satisfy two NOMA users’ different quality-of-service (QoS) requirements [14]. In [15], a two-way relay-based CNOMA (TW-CNOMA) scheme for DL and UL transmissions of two users was presented, in which FU is assisted by a dedicated relay node equipped with two antennas for simultaneous reception of bidirectional signals. Compared with conventional one-way relay-based CNOMA (OW-CNOMA) and OMA, works in [15] confirmed higher SE gain and a lower amount of required time slots. A power allocation scheme maximizing the achievable sum-rate was proposed in [16], where a relay node provides the ability to allow multiple FUs’ DL and UL transmissions in the TW-CNOMA scheme. Do and Van Nguyen studied NOMA systems with an AF/DF-aided BS-assisting communication from a wireless powered device to a nonenergy harvesting device, where two devices have distinct predefined QoS requirements [17]. In [18,19], energy harvesting (EH)-enabled CNOMA network accommodating two users, a source and an EH-assisted relay were studied under an imperfect channel state information (CSI) environment. In [20], a cognitive radio system employing NOMA technology was investigated, where the outage performance of the secondary network using relay selection policies was examined. NOMA was adopted in V2X services to reduce resource collision, by which the SE was improved and the latency was reduced [21]. It introduced a new paradigm to obtain high overloading transmission over limited resources [21].

Vehicular networks have opportunities as well as challenges, where solutions for challenges have been proposed in various forms in recent works [22,23]. In order to achieve low latency and high reliability in vehicular networks, NOMA was employed in various scenarios. A V2X broadcasting system utilizing NOMA was studied to provide a mixed, centralized/distributed architecture and to distribute power control to participating vehicles [23]. Khoueiry and Soleymani pointed out that the V2X network can achieve a rate close to the capacity bound by exploiting new graph-based practical encoding and joint belief propagation decoding techniques [24]. In [25], NOMA-spatial modulation (NOMA-SM), which benefits from the robustness of SM against channel correlation, was proposed to improve bandwidth efficiency and to deal with the harmful effects of wireless V2V environments. 5G V2X communications using two relay-assisted NOMA transmission schemes were studied in [26], in which optimal power allocations for full-duplex relay-assisted NOMA (FDR-NOMA) and half-duplex relay-assisted NOMA (HDR-NOMA) broadcasting and multicasting systems. A NOMA system employing power allocation algorithm with opportunistic constraints was proposed to improve the throughput of V2X networks [27]. A hierarchical power control method via dynamic power allocation from BSs to vehicles was introduced to optimize the energy efficiency of the vehicular networks [28].

Inspired by the above observations, we apply NOMA technology to 5G V2X communications and investigate the performance at each vehicle. We consider a system model conveying the problem of half-duplex relay-assisted broadcasting/multicasting for V2I. Different from [26], we consider the relay-assisted NOMA-based broadcasting/multicasting schemes for the links from BS to vehicles. We propose a resource management algorithm which is central and thus needs to be implemented at the BS. We add FDR-NOMA scheme to the result of [20] and compare it with HDR-NOMA. The proposed transmission schemes can be adopted in communications from infrastructure to vehicles. By integrating NOMA and relay-assisted broadcasting/multicasting communications, we can improve spectrum efficiency, increase the number of connections, and achieve lower latency and high reliability. The main contributions of this paper are listed below:

- We consider multi-antennas at vehicles. NOMA scheme is suitable for communications between two vehicles driving around the roadside units (RSU). In order to share information more efficiently, the communication mode of V2X will mainly be two-way transmission, rather than one-way connection.
- For the sake of expanding the network coverage, we use RSU to assist vehicles to communicate with BS. In the broadcasting scenario, each RSU transmits information to vehicles in different directions. Different from the traditional OMA scenario, RSU serves multiple vehicles using NOMA in both uplink and downlink scenarios, since vehicles in the same group need to receive different information from the RSU.
- We derive the exact outage probability expressions for two vehicles in a pair in terms of perfect SIC (pSIC) and imperfect SIC (ipSIC). The obtained closed-form expression for outage probability is quite accurate. The system throughput is also discussed in the mode of delay-limited transmission.
- Impacts of SIC operation at receivers in the context of NOMA are carefully considered. The system performance in terms of outage probability can be enhanced by increasing the number of transmit antennas equipped at vehicles.

The rest of this paper is organized as follows. In Section 2, the model of V2X communication employing NOMA and multiple antennas is introduced. In Section 3, the closed-form analyses for outage probability and throughput, which are the main performance metrics, are conducted. Computer simulations are performed in Section 4 and the conclusion is provided in Section 5.

**Notation**: Pr{·} stands for a probability. $f_{X}\left(\cdot \right)$, $F_{X}\left(\cdot \right)$, and E{·} represent the probability density function (pdf), the cumulative distribution function (CDF), and the expectation of a random variable *X*, respectively. Γ(·) denotes a Gamma function.

## 2. System Model of V2X Communications

**Figure 1:**
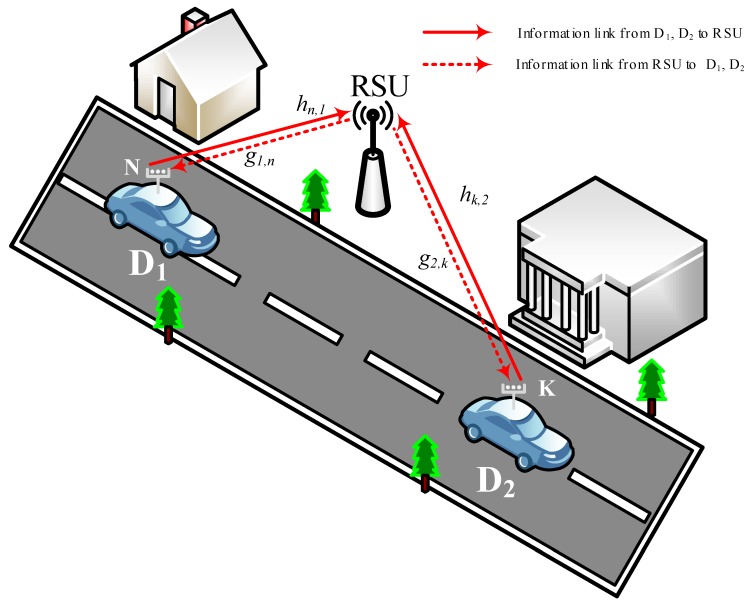
System model of Non-orthogonal multiple access (NOMA)-based vehicle-to-everything (V2X) systems.

We consider a two-way relay-assisted NOMA system as depicted in Figure 1. A pair of vehicles, $D_{1}$ and $D_{2}$, participate in two-way communications via the RSU following the principle of NOMA. Let $P_{s}$ denote the transmit power of vehicles $D_{1}$ and $D_{2}$, while $P_{r}$ denotes the transmit power of RSU. We suppose $P_{s}$ and $P_{r}$ are equal for a simple analysis, i.e., $P_{s}=P_{r}=P$. Let $x_{1}$ and $x_{2}$ denote the transmit signals with unit power of vehicles $D_{1}$ and $D_{2}$, respectively. The RSU plays the role of relay, which transfers the signal from $D_{1}$ to $D_{2}$ and vice versa within its coverage. The RSU operates in the decode-and-forward half-duplex relaying mode. Note that the proposed transmission scheme can be adopted in the communication from vehicle to vehicle via infrastructure node (relay node). We suppose that the RSU is able to share the same frequency resource with the BS. We also assume that the direct link between two vehicles is weak and ignored due to a large path loss. Vehicles are equipped with multiple antennas, where $D_{1}$ and $D_{2}$ have *N* antennas and *K* antennas, respectively. Channel gains between the *n*-th antenna of $D_{1}$ and RSU for uplink and downlink are denoted by $h_{n,1}$ and $g_{1,n}$, respectively, n=1,2,…,N. In the same manner, we denote $h_{k,2}$ and $g_{2,k}$, k=1,2,…,K, as channel gains between the *k*-th antenna of $D_{2}$ and RSU for uplink and downlink, respectively. We let $h_{n,1}$, n=1,⋯,N, and assume that they are independent and identically distributed (i.i.d.) Nakagami-m random variables with a fading parameter $m_{h_{1}}$. In the same manner, $h_{k,2}$, $g_{1,n}$, and $g_{2,k}$ are i.i.d. Nakagami-m distributed with fading parameters $m_{h_{2}}$, $m_{g_{1}}$, and $m_{g_{2}}$, respectively, for all *k* and *n*. We also let $\lambda_{h_{1}}=E\left\{{\left|h_{n,1}\right|}^{2}\right\}$, $\lambda_{h_{2}}=E\left\{{\left|h_{k,2}\right|}^{2}\right\}$ , $\lambda_{g_{1}}=E\left\{{\left|g_{1,n}\right|}^{2}\right\}$, and $\lambda_{g_{2}}=E\left\{{\left|g_{2,k}\right|}^{2}\right\}$ for all *n* and *k*. The residual interference signal (IS) caused by ipSIC is also modeled as a Nakagami-m random variable [29]. Let $f_{1}$ and $f_{2}$ denote the IS channel coefficients at the RSU and $D_{1}$, respectively, where $\lambda_{f_{1}}=E\left\{{\left|f_{1}\right|}^{2}\right\}$ and $\lambda_{f_{2}}=E\left\{{\left|f_{2}\right|}^{2}\right\}$. Then, we define $\mu_{q}=\frac{m_{q}}{\lambda_{q}}$, where $q=h_{1},h_{2},g_{1},g_{2},f_{1},f_{2}$.

The entire transmission process of NOMA-V2X is divided into two phases. In the first phase, vehicles $D_{1}$ and $D_{2}$ transmit signals $x_{1}$ and $x_{2}$, respectively, with the power of $P_{s}$ to the RSU. The RSU detects $x_{1}$ and $x_{2}$ through a SIC technique, where pSIC or ipSIC are considered. After detecting $x_{1}$, the RSU subtracts $x_{1}$ from the received signal and perform the next transmission hop. In the second phase, the RSU transmits the superimposed NOMA signal, combining two signals $x_{1}$ and $x_{2}$ to vehicles $D_{1}$ and $D_{2}$, where $P_{r}$ is divided by the ratio of $a_{1}:a_{2}$ to be used for transmitting NOMA signal to $D_{2}$ and $D_{1}$, respectively. In other words, the RSU uses $\frac{a_{1}}{a_{1}+a_{2}}P_{r}$ and $\frac{a_{2}}{a_{1}+a_{2}}P_{r}$ to transmit signals $x_{1}$ and $x_{2}$, respectively. We suppose $D_{2}$ is farther away from the RSU than $D_{1}$, by which the link between $D_{1}$ and RSU is stronger than that between $D_{2}$ and RSU. Since higher transmit power is required for the vehicle with a weaker link, we let $a_{1}>a_{2}$ in our model.

Suppose that each vehicle chooses one antenna having the highest channel gain among multiple antennas to communicate with RSU. In the first phase, the signal to interference plus noise ratio (SINR) experienced by the RSU when detecting $x_{1}$ sent from $D_{1}$ and $x_{2}$ sent from $D_{2}$ are obtained by

(1) $$\begin{array}{c} \gamma_{D_{1}\to R}=\frac{{\left|h_{n^{*},1}\right|}^{2}\rho }{{\left|h_{k^{*},2}\right|}^{2}\rho +1} \end{array}$$

and

(2) $$\begin{array}{c} \gamma_{D_{2}\to R}=\frac{{\left|h_{k^{*},2}\right|}^{2}\rho }{{\left|f_{1}\right|}^{2}\varpi \rho +1}, \end{array}$$

respectively, where $\rho =\frac{P}{N_{0}}$ is the common signal-to-noise ratio (SNR) without fading and $f_{1}$ denotes the IS channel coefficient at the RSU. Here, ϖ=0 and ϖ=1 indicate that pSIC and ipSIC is used at the receiver, respectively, and $n^{*}={\mathrm{argmax}}_{1\leq n\leq N}{\left|h_{n,1}\right|}^{2}$, $k^{*}={\mathrm{argmax}}_{1\leq n\leq K}{\left|h_{k,2}\right|}^{2}$.

In the second phase, two vehicles further process the received NOMA signals sent from the RSU. Note that the signal $x_{1}$ has a higher priority than $x_{2}$ under the condition of $a_{1}>a_{2}$. The SINR at $D_{2}$ when detecting $x_{1}$ is written as

(3) $$\gamma_{D_{2},x_{1}}=\frac{{\left|g_{2,k^{*}}\right|}^{2}\rho a_{1}}{{\left|g_{2,k^{*}}\right|}^{2}\rho a_{2}+1},$$

where $k^{*}={\mathrm{argmax}}_{1\leq k\leq K}{\left|g_{2,k}\right|}^{2}$. According to NOMA, the SIC algorithm is adopted at $D_{1}$ as well. Then, the SINR at $D_{1}$ for detecting $x_{1}$ is given by

(4) $$\gamma_{D_{1},x_{1}}=\frac{{\left|g_{1,n^{*}}\right|}^{2}\rho a_{1}}{{\left|g_{1,n^{*}}\right|}^{2}\rho a_{2}+1}$$

and the SINR at $D_{1}$ for detecting $x_{2}$ is written as

(5) $$\gamma_{D_{1},x_{2}}=\frac{{\left|g_{1,n^{*}}\right|}^{2}\rho a_{2}}{{\left|f_{2}\right|}^{2}\varpi \rho +1},$$

where $n^{*}={\mathrm{argmax}}_{1\leq n\leq N}{\left|g_{1,n}\right|}^{2}$ and $f_{2}$ denotes the IS channel coefficients at $D_{1}$.

It is expected from the above analysis that the received SINR depends on instantaneous channel gains and power allocation ratio $a_{1}:a_{2}$. The communication process of V2X is affected by the number of antennas implemented at each vehicle, although the ability of receivers is related to which SIC is used between pSIC or ipSIC. It is predicted that different performances are obtained by considering different SIC, i.e., pSIC or ipSIC, different qualities for channels, and different numbers of antennas.

## 3. Analysis of Outage Probability and Throughput of NOMA-V2X System

In this section, the outage probability and throughput of the NOMA-V2X system are investigated as performance measures. Requirements for outage performance and QoS of vehicles can be satisfied in the NOMA-V2X system. The outage probability is examined to check the performance of vehicles over Nakagami-m fading channels. Especially, we analyze NOMA-V2X network by considering multiple antennas at vehicles for different scenarios. Let $R_{1}$ and $R_{2}$ denote the predetermined target rates of $D_{1}$ and $D_{2}$, respectively. In principle, an outage occurs when the achievable rate is smaller than the predetermined target rate.

### 3.1. Outage probability of $D_{2}$

We let *Z* denote a random variable representing $f_{1}$ and $f_{2}$. Then, the CDF and pdf of *Z* are defined by

(6) $$F_{Z}\left(x\right)=1-e^{-\mu_{z}x}\sum_{s=0}^{m_{z}-1}\frac{{\left(\mu_{z}x\right)}^{s}}{s!}$$

and

(7) $$f_{Z}\left(x\right)=\frac{\mu_{z}^{m_{z}}x^{m_{z}-1}e^{-\mu_{z}x}}{\Gamma \left(m_{z}\right)},$$

respectively, where $\mu_{z}=\frac{m_{z}}{\lambda_{z}}$ is a parameter of multipath fading associated with $f_{1}$ and $f_{2}$. Let *Y* denote the channel gain corresponding to the best antenna of $D_{1}$ or $D_{2}$ selected for communication in V2X systems. The CDF and pdf of *Y* are defined by

(8) $$F_{Y}\left(x\right)={\left[1-e^{-\mu_{y}x}\sum_{t=0}^{m_{y}-1}\frac{{\left(\mu_{y}x\right)}^{t}}{t!}\right]}^{G}$$

and

(9) $$f_{Y}\left(x\right)=\frac{G\mu_{y}^{m_{y}}x^{m_{y}-1}e^{-\mu_{y}x}}{\Gamma \left(m_{y}\right)}\times \underset{\overset{\Delta }{\;=}\Theta }{\underset{︸}{{\left[1-e^{-\mu_{y}x}\sum_{t=0}^{m_{y}-1}\frac{{\left(\mu_{y}x\right)}^{t}}{t!}\right]}^{G-1}}},$$

respectively, where $\mu_{y}=\frac{m_{y}}{\lambda_{y}}$ and $\Theta ≜{\left(1-e^{-\mu_{y}x}\sum_{t=0}^{m_{y}-1}\frac{{\left(\mu_{y}x\right)}^{t}}{t!}\right)}^{G-1}$. Note that G=N and G=K if *Y* is associated with $D_{1}$ and $D_{2}$, respectively. By applying a successive binomial expansion to (8), we obtain

(10) FYx=∑i=0G⋃i′Gi−1iAi′Bi′xi¯e−μyix,

where $\underset{i^{\prime }}{⋃}\overset{\Delta }{\;=}\sum_{i_{1}=0}^{i}\sum_{i_{2}=0}^{i-i_{1}}\cdots \sum_{i_{m_{z}-1}=0}^{i-i_{1}-\cdots -i_{m_{z}-2}}$, Ai′=ii1i−i1i2⋯i−i1−⋯−imz−2imz−1, $B_{i^{\prime }}=\prod_{t=0}^{m_{z}-2}{\left(\frac{\mu_{z}^{t}}{t!}\right)}^{i_{t}+1}{\left(\frac{\mu_{z}^{m_{z}-1}}{\Gamma \left(m_{z}\right)!}\right)}^{i-i_{1}-i_{2}-\cdots -i_{m_{z}-1}}$ and $\bar{i}=\left(m_{z}-1\right)\left(i-i_{1}\right)-\left(m_{z}-2\right)i_{2}-\cdots -i_{m_{z}-1}$. We also apply the successive binomial expansion to Θ to obtain

(11) Θ=∑j=0G−1⋃j′G−1j−1jAj′Bj′xj¯e−μyjx.

Let us consider the link $D_{1}\to \mathrm{RSU}\to D_{2}$ and suppose that RSU decodes $x_{1}$ correctly in the first phase and $D_{2}$ also decodes $x_{1}$ correctly in the second phase. Note that RSU can decode $x_{2}$ after decoding $x_{1}$ correctly. Since $x_{1}$ is decoded first at RSU before using SIC, the outage probability for $D_{1}\to \mathrm{RSU}\to D_{2}$ link does not depend on SIC. Thus, the outage probabilities of $D_{2}$ obtained by using pSIC and ipSIC for $D_{1}\to \mathrm{RSU}\to D_{2}$ link are the same and they are defined as

(12) $$P_{2}^{pSIC}=P_{2}^{ipSIC}≜P_{2}^{SIC}=1-\mathrm{Pr}\left\{\gamma_{D_{1}\to R}>\gamma_{2},\gamma_{D_{2},x_{1}}>\gamma_{2}\right\},$$

which is expanded as

(13) P2SIC=1−∑j=0K−1∑l=0K⋃l′⋃j′KlK−1jKμh2mh2−1j+lAj′Bj′Al′Bl′χl¯δl¯e−μg2lχδΓmh2×Γmh2+j¯μh2j¯1+jmh2+j¯−∑i=0N∑q=0i¯⋃i′i¯qNi−1iρi¯−qδi¯Ai′Bi′Γmh2+j¯+i¯−qe−μh1iδμh21+j+μh1iδρmh2+j¯+i¯−q,

where $\gamma_{2}=2^{2R_{2}}-1$, $\delta =\frac{\gamma_{2}}{\rho }$ and $\chi =\frac{1}{\left(a_{1}-a_{2}\gamma_{2}\right)}$. The detailed derivation for (13) is given in Appendix A.

### 3.2. Outage Probability of $D_{1}$

The successful communication through $D_{2}\to \mathrm{RSU}\to D_{1}$ link is possible only if the following conditions are all met: i) RSU decodes $x_{1}$ correctly, ii) RSU decodes $x_{2}$ correctly by SIC, iii) $D_{1}$ decodes $x_{1}$ correctly, and iv) $D_{1}$ decodes $x_{2}$ correctly by SIC. Based on the category of SIC in NOMA, i.e., pSIC and ipSIC, different events of outage for $D_{1}$ can occur. Thus, the outage probability for detecting $x_{1}$ at the vehicle $D_{1}$ can be expressed in two distinct forms depending on which SIC scheme is used. In particular, ipSIC is considered as a main reason for degraded performance in term of outage probability.

First, we consider a worse case resulting in higher outage probability, in which the first vehicle $D_{1}$ utilizes ipSIC. This corresponds to the case of ϖ=1, and the outage probability of $D_{1}$, denoted by $P_{1}^{ipSIC}$, is defined and expanded as

(14) $$\begin{array}{cc} P_{1}^{ipSIC} & =1-\mathrm{Pr}\left\{\gamma_{D_{1}\to R}>\gamma_{1},\gamma_{D_{2}\to R}>\gamma_{2},\gamma_{D_{1},x_{1}}>\gamma_{1},\gamma_{D_{1},x_{2}}>\gamma_{2}\right\}\\ \; & =1-\mathrm{Pr}\left\{{\left|h_{n^{*},1}\right|}^{2}>\delta \left({\left|h_{k^{*},2}\right|}^{2}\rho +1\right),{\left|h_{k^{*},2}\right|}^{2}>\bar{\delta }\left({\left|f_{1}\right|}^{2}\varpi \rho +1\right)\right\}\\ \; & \;\times \mathrm{Pr}\left\{{\left|g_{1,n^{*}}\right|}^{2}>\delta \chi ,{\left|g_{1,n^{*}}\right|}^{2}>\bar{\phi }\left({\left|f_{2}\right|}^{2}\varpi \rho +1\right)\right\}, \end{array}$$

where $\gamma_{1}=2^{2R_{1}}-1$, $\gamma_{2}=2^{2R_{2}}-1$, $\delta =\frac{\gamma_{2}}{\rho }$, $\bar{\delta }=\frac{\gamma_{1}}{\rho }$, $\phi =\frac{\gamma_{2}}{\rho a_{1}}$, and $\bar{\phi }=\frac{\gamma_{1}}{\rho a_{2}}$. We expand (14) further as

(15) P1ipSIC=1−∑j=0K−1⋃j′∑r=0mh2+j¯−1∑t=0rrtK−1jΓmh2+j¯r!Γmh2Γmf1×Γmf1+r−tδ¯rNμh2mh2μf1mf1−1jAj′Bj′e−δ¯μh21+jμh2mh2+j¯−r1+jmh2+j¯−rμf1+δ¯ϖρμh21+jmf1+r−t−∑j=0M−1∑i=0N∑a=0i¯∑s=0mh2+j¯+i¯−a−1∑d=0s⋃i′⋃j′sdi¯aNiK−1j×KΓmh2+j¯+i¯−aΓmf1+s−dδ¯sμh2mh2μf1mf1ϖs−ds!Γmh2Γmf1μh21+j+μh1iδρmh2+j¯+i¯−a−s×−1j+iδi¯Aj′Bj′Ai′Bi′e−μh1iδ−δ¯μh21+j+μh1iδρρd+a−i¯−sμf1+δ¯ϖρμh21+j+μh1iδρmf1+s−d×∑k=0N⋃k′Nk−1kAk′Bk′δk¯χk¯e−μg1kδχΓmf2γmf2,Φμf2+∑k=0N∑c=0k¯⋃k′k¯cNk−1kϖcρcAk′Bk′μ2mf2ϕ¯k¯e−μg1kϕ¯Γmf2μf2+μg1kϕ¯ϖρmmf2+c×Γmmf2+c,μf2+μg1kϕ¯ϖρΦ,

whose detailed derivation is provided in Appendix B.

Second, we consider an ideal case resulting in a lower outage probability, in which the receiver of $D_{1}$ utilizes pSIC. This corresponds to ϖ=0, and the outage probability of the first vehicle, denoted by $P_{1}^{pSIC}$, is defined and expanded as

(16) $$\begin{array}{cc} P_{1}^{pSIC} & =1-\mathrm{Pr}\left\{\gamma_{D_{1}\to R}>\gamma_{1},\gamma_{D_{2}\to R}>\gamma_{2},\gamma_{D_{1},x_{1}}>\gamma_{1},\gamma_{D_{1},x_{2}}>\gamma_{2}\right\}\\ \; & =1-\mathrm{Pr}\left\{\gamma_{D_{1}\to R}>\gamma_{1},\gamma_{D_{2}\to R}>\gamma_{2}\right\}\times \mathrm{Pr}\left\{\gamma_{D_{1},x_{1}}>\gamma_{1},\gamma_{D_{1},x_{2}}>\gamma_{2}\right\}\\ \; & =1-\mathrm{Pr}\left\{{\left|h_{n^{*},1}\right|}^{2}>\delta \left({\left|h_{k^{*},2}\right|}^{2}\rho +1\right),{\left|h_{k^{*},2}\right|}^{2}>\bar{\delta }\right\}\times \mathrm{Pr}\left\{{\left|g_{1,n^{*}}\right|}^{2}>\Lambda \right\}, \end{array}$$

where $\Lambda =\max\left(\delta \chi ,\bar{\phi }\right)$. We further expand (16) as

(17) P1pSIC=1−∑j=0K−1∑r=0j¯+mh2−1⋃j′K−1jK−1jδ¯rΓj¯+mh2Aj′Bj′r!Γmh2μh2j¯−rj+1j¯+mh2−re−δ¯μh2j+1−∑j=0K−1∑i=0N∑a=0i¯∑s=0j¯+mh2+i¯−a−1⋃i′⋃j′jaNiK−1j×Kμh2mh2−1j+iΓj¯+mh2+i¯−aδi¯δ¯sρi¯−aAj′Bj′Ai′Bi′e−μh1iδ−δ¯μh2j+1+μh1iδρs!Γmh2μh2j+1+μh1iδρj¯+mh2+i¯−a−s×1−∑k=0N⋃k′Nk−1kAk′Bk′Λk¯e−μg1kΛ,

whose detailed derivation is given in Appendix C.

**Remark** **1.** *Based on above analytical results of outage performance at each vehicle, it is inferred that the system performance depends on what kind of SIC (i.e., ipSIC or pSIC) is used at the receiver. The higher outage probability is obtained for V2X-NOMA with ipSIC due to the existence of residual interference in SINR.*

### 3.3. Throughput

The throughput of the system indicates how much NOMA-V2X is able to enable transmission in the context of vehicles. The outage throughput of vehicles $D_{1}$ and $D_{2}$ is defined with the fixed transmission rates $R_{1}$ and $R_{2}$, respectively, as

(18) $$\begin{array}{cc} \tau_{⋆}^{\theta }=\left(1-P_{⋆}^{\theta }\right)R_{⋆} & ,\left\{\begin{array}{c} ⋆\in \left\{1,2\right\}\\ \theta \in \left\{pSIC,ipSIC\right\} \end{array}\right.. \end{array}$$

## 4. Numerical Results

For performance evaluation of NOMA-V2X, we investigate a typical pair of vehicles with random pairing in NOMA networks. We perform Monte Carlo simulations to obtain outage probabilities of vehicles with the following parameters. Note that chosen parameters are similar to those used in [30]. Target data rates for vehicles $D_{1}$ and $D_{2}$ are set as $R_{1}=3$ [BPCU] and $R_{2}=1$ [BPCU], respectively. Without loss of generality, the power allocation ratio for transmitting $x_{1}$ and $x_{2}$ at RSU is set as $a_{1}=0.8$ and $a_{2}=0.2$. We suppose Nakagami-m distributed channel gains $h_{n^{*},1}$ and $g_{1,n^{*}}$ with $\lambda_{h_{1}}=\lambda_{g_{1}}=d^{-\alpha }$, and $h_{k^{*},2}$ and $g_{2,k^{*}}$ with $\lambda_{h_{2}}=\lambda_{g_{2}}={\left(1-d\right)}^{-\alpha }$, where α=2 and d=0.3 are considered. We also consider $\lambda_{f_{1}}=\lambda_{f_{2}}=-10$ [dB]. We let all channels have the same fading parameters, i.e., $m=m_{h_{1}}=m_{h_{2}}=m_{g_{1}}=m_{g_{2}}=m_{f_{1}}=m_{f_{2}}$. Note that other remaining key parameters are specified in simulation results.

**Figure 2:**
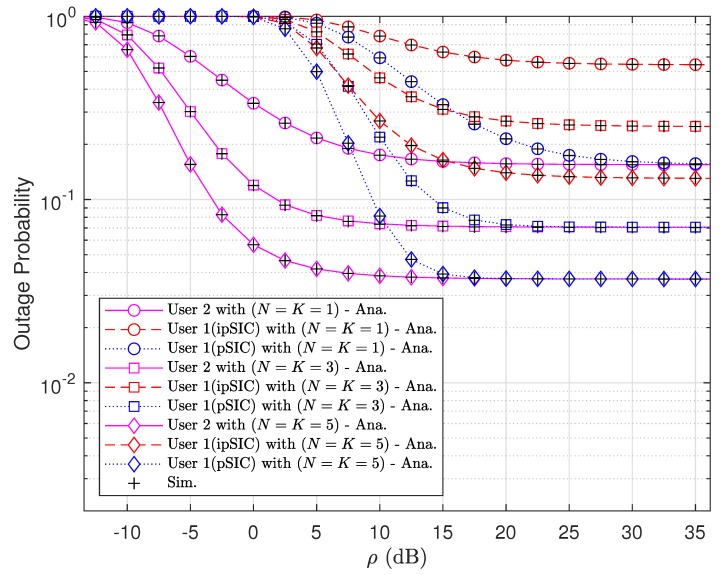
Outage probabilities of two vehicles versus transmit signal-to-noise ratio (SNR) with a fading parameter m=1.

Figure 2 shows how the increase of transmit power at the RSU influences the performance of NOMA-V2X system in term of outage probability, where various numbers of antennas at vehicles are considered. We use the same number of antennas for $D_{1}$ and $D_{2}$, i.e., N=K, where N=K=1,3,5 are considered. Since a better channel can be selected more probably with a higher number of antennas at vehicles, the lowest outage probability can be observed for the second vehicle $D_{2}$ with N=K=5. It is observed that the outage probability of two vehicles are considerably different at low transmit power, i.e., ρ<15 [dB], while the outage performance of two vehicles are similar at high transmit power regime. It is also observed that, in general, $D_{2}$ has lower outage probability than $D_{1}$. Note that we obtain lower outage probability over all ranges of transmit SNR by using pSIC at $D_{1}$.

**Figure 3:**
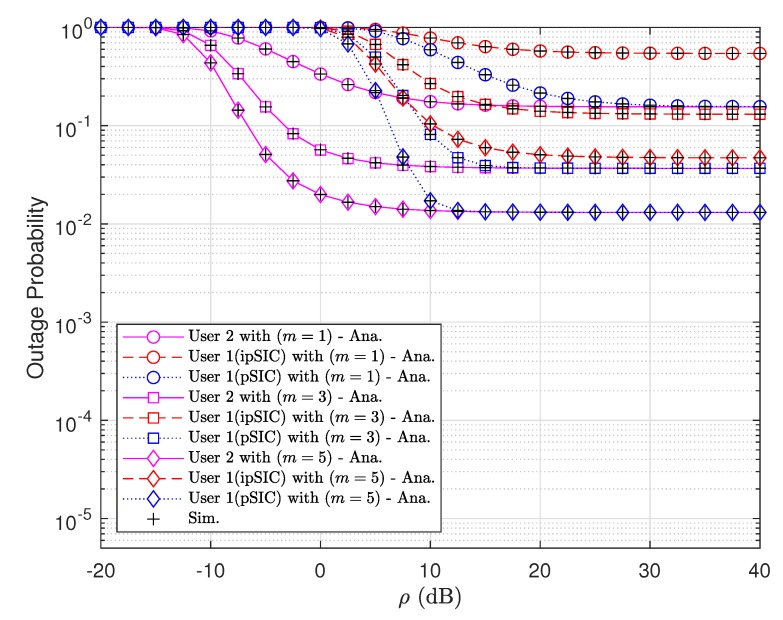
Outage probability obtained for various fading parameters *m* with N=K=3.

In Figure 3, we plot outage probabilities obtained theoretically versus transmit SNR ρ with the fixed number of antennas at two vehicles. The lowest outage probabilities of two vehicles are obtained with pSIC condition and a fading parameter m=5, where different fading parameters result in different outage performance. In case of using pSIC at the first vehicle $D_{1}$, the cooperative NOMA system under consideration results in the same outage probability for both vehicles at high SNR, i.e., ρ>12 [dB].

**Figure 4:**
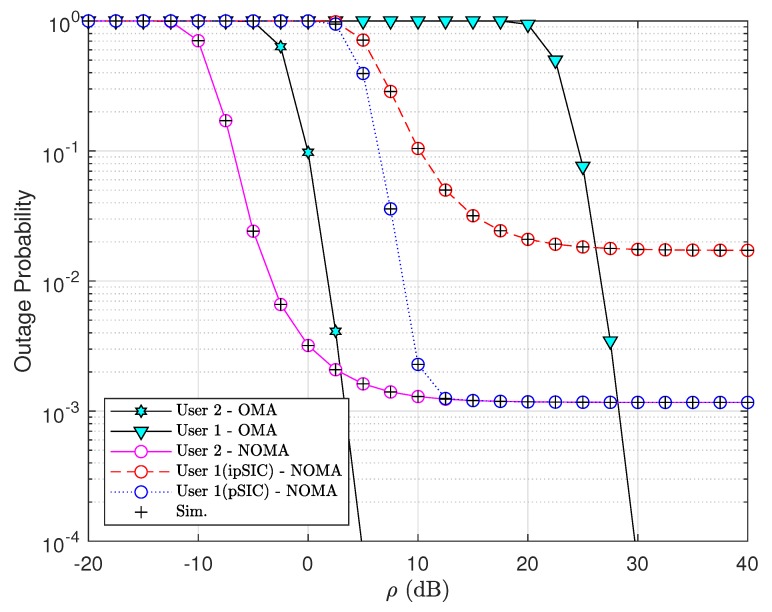
Comparison between NOMA-V2X and orthogonal multiple access (OMA)-V2X regarding outage performance of two vehicles with N=K=3 and m=3.

In Figure 4, we plot outage probabilities of NOMA-V2X and OMA-V2X systems obtained analytically and by simulations with pSIC and ipSIC at the first vehicle. The outage performance comparison is presented to illustrate the outage alleviation occurring at specific range of ρ. Note that theoretical evaluations match well with simulation results. All other trends of the outage probabilities of two vehicles are similar to previous results.

**Figure 5:**
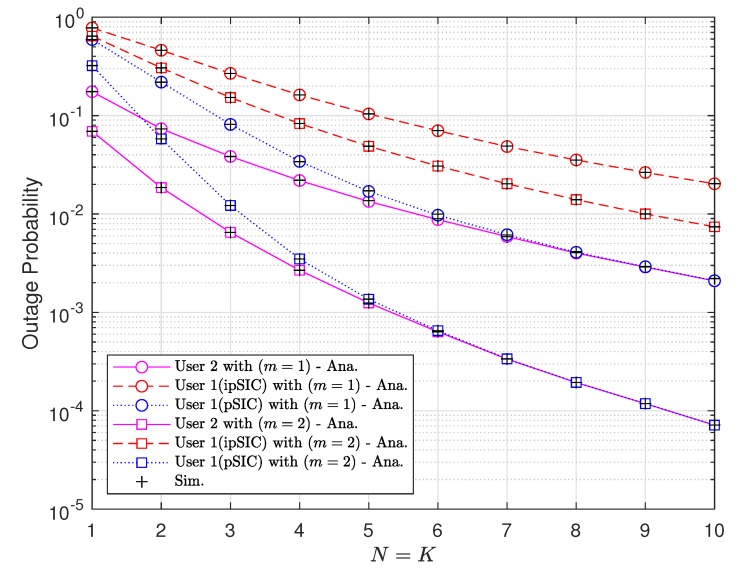
Outage probabilities with respect to the number of antennas at each vehicle with ρ=10 [dB].

Figure 5 shows the outage probability with respect to the number of antennas at each vehicle with perfect and imperfect SIC, where two fading parameters m=1 and 2 are considered. With the pSIC at $D_{1}$, the outage performance can be improved tremendously by increasing the number of antennas. The gap between the outage performances of $D_{1}$ obtained with pSIC and ipSIC gets more considerable as the number of antennas grows. In general, the outage performance of NOMA-V2X can be improved by using a higher number of antennas because using more antennas results in higher possibility of choosing the better channel and thus have higher SINR. Note that higher SINR leads to the higher probability of having perfect SIC operation and higher fading parameter *m*.

**Figure 6:**
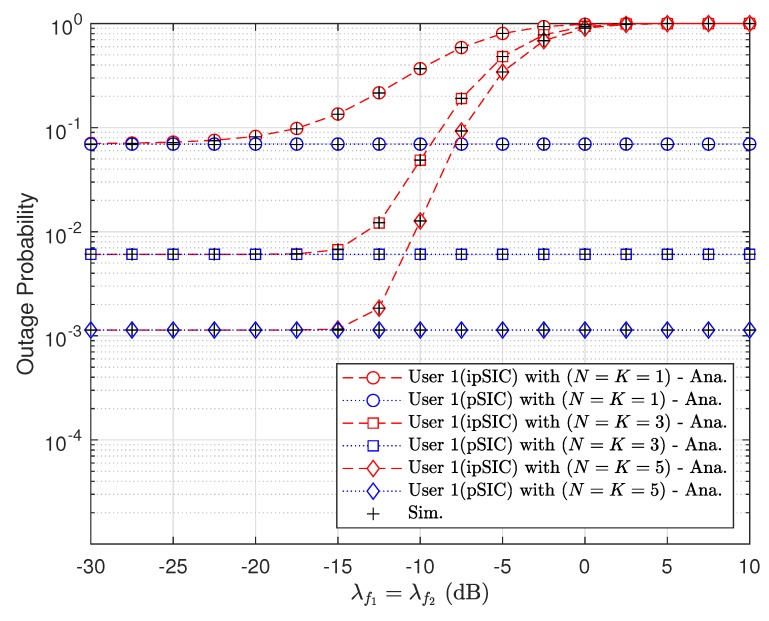
Impact of imperfect successive interference cancellation (SIC) levels to outage performance at the first vehicle with m=3 and ρ=20 [dB].

Figure 6 depicts the impact of imperfect SIC on the outage behavior, where the signal detection at $D_{1}$ is performed through SIC operation. The higher values of $\lambda_{f_{1}}$ and $\lambda_{f_{2}}$ result in the higher outage probability because they lead to smaller values of SINR.

**Figure 7:**
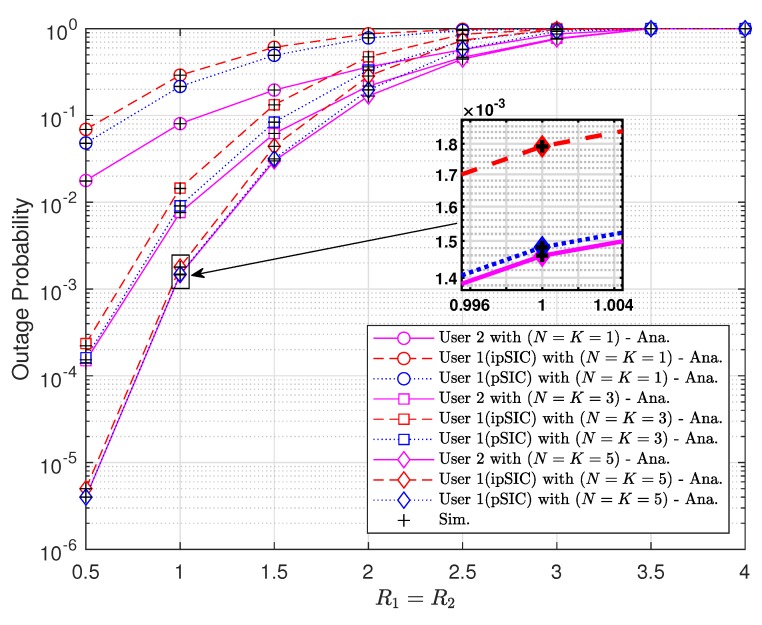
Outage probabilities versus target data rates with $a_{1}=0.9$, $a_{2}=0.1$, m=2, and ρ=5 [dB].

**Figure 8:**
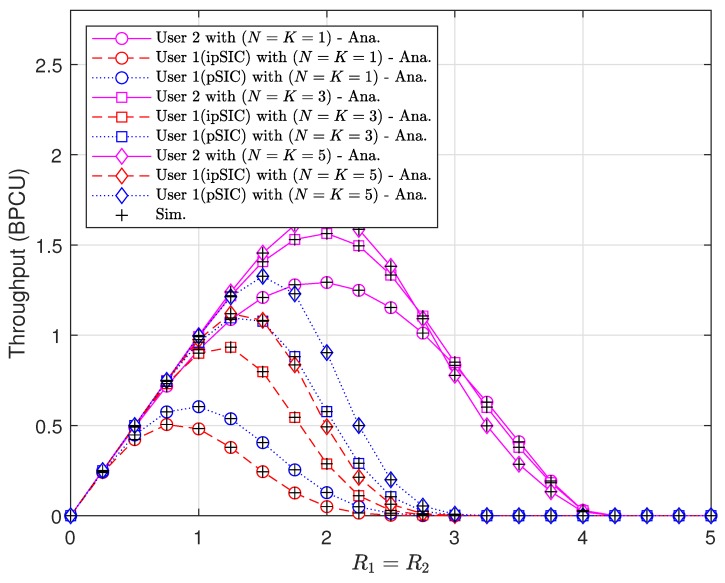
Throughput performance versus target data rates with $a_{1}=0.95$, $a_{2}=0.05$, m=2, and ρ=5 [dB].

It is observed from Figure 7 that higher target rates result in higher outage probabilities of two vehicles. However, the throughput of vehicles does not grow as target rates get higher as shown in Figure 8. The maximum throughput is obtained at medium values of target rates. The values of rates maximizing the throughput depend on the number of antennas and SIC scheme.

## 5. Conclusions

In this paper, we studied the outage performance of vehicles equipped with multiple antennas in NOMA-V2X networks over Nakagami-m fading channels. We derived closed-form expressions for outage probabilities of vehicles considering two different SIC operations, i.e., pSIC and ipSIC. Based on this result, we investigated the impacts of various design parameters to the outage performance of the NOMA-V2X system in various aspects. This investigation provides the theoretical guidelines for the actual design of the NOMA-V2X communication system. Analyses and simulations demonstrate that NOMA-V2X can achieve significant outage performance improvement with a high fading parameter, a high number of antennas, and a perfect SIC operation. In addition, we found that the limiting impact of an imperfect SIC can keep outage performance at an acceptable level in the real applications of NOMA-V2X system.

## Appendix A. Derivation of (13)

We rewrite (12) as

(A1) $$\begin{array}{cc} P_{2}^{SIC}\; & =1-\mathrm{Pr}\left\{\gamma_{D_{1}\to R}>\gamma_{2},\gamma_{D_{2},x_{1}}>\gamma_{2}\right\}\\ \; & =1-\underset{\overset{\Delta }{\;=}\varsigma_{1}}{\underset{︸}{\mathrm{Pr}\left\{{\left|h_{n^{*},1}\right|}^{2}>\delta \left({\left|h_{k^{*},2}\right|}^{2}\rho +1\right)\right\}}}\underset{\overset{\Delta }{\;=}\varsigma_{2}}{\underset{︸}{\mathrm{Pr}\left\{{\left|g_{2,k^{*}}\right|}^{2}>\chi \delta \right\}}}. \end{array}$$

Note that $\varsigma_{1}$ is defined and expanded as

(A2) $$\begin{array}{cc} \varsigma_{1} & ≜\mathrm{Pr}\left\{{\left|h_{n^{*},1}\right|}^{2}>\delta \left({\left|h_{k^{*},2}\right|}^{2}\rho +1\right)\right\}\\ \; & =\int_{0}^{\infty }f_{{\left|h_{k^{*},2}\right|}^{2}}\left(x\right)\left[1-F_{{\left|h_{n^{*},1}\right|}^{2}}\left(\delta \left(x\rho +1\right)\right)\right]dx\\ \; & =\underset{\overset{\Delta }{\;=}\varsigma_{1,1}}{\underset{︸}{\int_{0}^{\infty }f_{{\left|h_{k^{*},2}\right|}^{2}}\left(x\right)dx}}-\underset{\overset{\Delta }{\;=}\varsigma_{1,2}}{\underset{︸}{\int_{0}^{\infty }f_{{\left|h_{k^{*},2}\right|}^{2}}\left(x\right)F_{{\left|h_{n^{*},1}\right|}^{2}}\left(\delta \left(x\rho +1\right)\right)dx}}, \end{array}$$

where

(A3) ς1,1=∫0∞fhk*,22xdx=∑j=0M−1⋃j′M−1jMμh2mh2−1jAj′Bj′Γmh2∫0∞xmh2+j¯−1e−xμh21+jdx=∑j=0K−1⋃j′K−1jKμh2mh2−1jAj′Bj′Γmh2+j¯Γmh2μh2j¯1+jmh2+j¯,

and

(A4) ς1,2=∫0∞fhk*,22xFhn*,12δxρ+1dx=∑j=0K−1∑i=0N⋃i′⋃j′NiK−1jKμh2mh2−1j+iδi¯Aj′Bj′Ai′Bi′e−μh1iδΓmh2×∫0∞xmh2+j¯−1xρ+1i¯e−xμh21+j+μh1iδρdx.

Note that the last equality of (A3) is obtained by some manipulations based on (3.381.4) in [31]; and the last equality of (A4) is obtained by using a trinomial expansion (1.111) in [31]. By some manipulations based on (3.381.4) in [31], (A4) can be further expanded as

(A5) ς1,2=∑j=0K−1∑i=0N∑q=0i¯⋃i′⋃j′i¯qNiK−1jKμh2mh2−1j+iρi¯−qδi¯Aj′Bj′Ai′Bi′e−μh1iδΓmh2×∫0∞xmh2+j¯+i¯−q−1e−xμh21+j+μh1iδρdx=∑j=0K−1∑i=0N∑q=0i¯⋃i′⋃j′i¯qNiK−1jKμh2mh2−1j+iρi¯−qδi¯Aj′Bj′Ai′Bi′Γmh2+j¯+i¯−qe−μh1iδΓmh2μh21+j+μh1iδρmh2+j¯+i¯−q.

Consequently, (A2) is written as

(A6) ς1=∑j=0K−1⋃j′K−1jKμh2mh2−1jAj′Bj′Γmh2×Γmh2+j¯μh2j¯1+jmh2+j¯−∑i=0N∑q=0i¯⋃i′i¯qNi−1iρi¯−qδi¯Ai′Bi′Γmh2+j¯+i¯−qe−μh1iδμh21+j+μh1iδρmh2+j¯+i¯−q.

In the similar manner, $\varsigma_{2}$ is defined and expanded as

(A7) ς2≜Prg2,k*2>χδ=Fg2,k*2χδ=∑l=0K⋃l′Kl−1lAl′Bl′χl¯δl¯e−μg2lχδ.

Plugging (A6) and (A7) into (A1), we obtain

(A8) P2SIC=1−∑j=0K−1∑l=0K⋃l′⋃j′KlK−1jKμh2mh2−1j+lAj′Bj′Al′Bl′χl¯δl¯e−μg2lχδΓmh2×Γmh2+j¯μh2j¯1+jmh2+j¯−∑i=0N∑q=0i¯⋃i′i¯qNi−1iρi¯−qδi¯Ai′Bi′Γmh2+j¯+i¯−qe−μh1iδμh21+j+μh1iδρmh2+j¯+i¯−q,

which is (13).

## Appendix B. Derivation of (15)

We write (14) as

(A9) $$\begin{array}{cc} P_{1}^{ipSIC}\; & =1-\underset{\overset{\Delta }{\;=}\varsigma_{3}}{\underset{︸}{\mathrm{Pr}\left\{{\left|h_{n^{*},1}\right|}^{2}>\delta \left({\left|h_{k^{*},2}\right|}^{2}\rho +1\right),{\left|h_{k^{*},2}\right|}^{2}>\bar{\delta }\left({\left|f_{1}\right|}^{2}\varpi \rho +1\right)\right\}}}\\ \; & \;\times \underset{\overset{\Delta }{\;=}\varsigma_{4}}{\underset{︸}{\mathrm{Pr}\left\{{\left|g_{1,n^{*}}\right|}^{2}>\delta \chi ,{\left|g_{1,n^{*}}\right|}^{2}>\bar{\phi }\left({\left|f_{2}\right|}^{2}\varpi \rho +1\right)\right\}}}. \end{array}\;$$

Note that $\varsigma_{3}$ is defined and expanded as

(A10) $$\begin{array}{cc} \varsigma_{3} & =\mathrm{Pr}\left\{{\left|h_{n^{*},1}\right|}^{2}>\delta \left({\left|h_{k^{*},2}\right|}^{2}\rho +1\right),{\left|h_{k^{*},2}\right|}^{2}>\bar{\delta }\left({\left|f_{1}\right|}^{2}\varpi \rho +1\right)\right\}\\ \; & =\int_{0}^{\infty }f_{{\left|f_{1}\right|}^{2}}\left(x\right)\int_{\bar{\delta }\left(x\varpi \rho +1\right)}^{\infty }f_{{\left|h_{k^{*},2}\right|}^{2}}\left(y\right)\left[1-F_{{\left|h_{n^{*},1}\right|}^{2}}\left(\delta \left(y\rho +1\right)\right)\right]dx\\ \; & =\underset{\overset{\Delta }{\;=}\varsigma_{3,1}}{\underset{︸}{\int_{0}^{\infty }\int_{\bar{\delta }\left(x\varpi \rho +1\right)}^{\infty }f_{{\left|f_{1}\right|}^{2}}\left(x\right)f_{{\left|h_{k^{*},2}\right|}^{2}}\left(y\right)dxdy}}\\ \; & \;-\underset{\overset{\Delta }{\;=}\varsigma_{3,2}}{\underset{︸}{\int_{0}^{\infty }\int_{\bar{\delta }\left(x\varpi \rho +1\right)}^{\infty }f_{{\left|f_{1}\right|}^{2}}\left(x\right)f_{{\left|h_{k^{*},2}\right|}^{2}}\left(y\right)F_{{\left|h_{n^{*},1}\right|}^{2}}\left(\delta \left(y\rho +1\right)\right)dxdy}}, \end{array}\;$$

where

(A11) ς3,1=∫0∞∫δ¯xϖρ+1∞ff12xfhk*,22ydxdy=∑j=0K−1⋃j′K−1jKμh2mh2μf1mf1−1jAj′Bj′Γmh2Γmf1∫0∞xmf1−1e−μf1x∫δ¯xϖρ+1∞ymh2+j¯−1e−yμh21+jdxdy=∑j=0K−1⋃j′∑r=0mh2+j¯−1K−1j∫0∞xmf1−1e−xμf1+δ¯ϖρμh21+jxϖρ+1rdx×Γmh2+j¯δ¯rKμh2mh2μf1mf1−1jAj′Bj′e−δ¯μh21+jr!Γmh2Γmf1μh2mh2+j¯−r1+jmh2+j¯−r=∑j=0K−1⋃j′∑r=0mh2+j¯−1∑t=0rrtK−1j∫0∞xmf1+r−t−1e−xμf1+δ¯ϖρμh21+jdx×Γmh2+j¯δ¯rKμh2mh2μf1mf1−1jAj′Bj′e−δ¯μh21+jr!Γmh2Γmf1μh2mh2+j¯−r1+jmh2+j¯−r=∑j=0K−1⋃j′∑r=0mh2+j¯−1∑t=0rrtK−1j×Γmh2+j¯Γmf1+r−tδ¯rKμh2mh2μf1mf1−1jAj′Bj′e−δ¯μh21+jr!Γmh2Γmf1μh2mh2+j¯−r1+jmh2+j¯−rμf1+δ¯ϖρμh21+jmf1+r−t

and

(A12) ς3,2=∫0∞∫δ¯xϖρ+1∞ff12xfhk*,22yFhn*,12δyρ+1dxdymf1=∑j=0K−1∑i=0N⋃i′⋃j′NiK−1jKμh2mh2μf1mf1−1j+iδi¯Aj′Bj′Ai′Bi′e−μh1iδΓmh2Γmf1×∫0∞xmf1−1e−μf1x∫δ¯xϖρ+1∞ymh2+j¯−1e−yμh21+j+μh1iδρyρ+1i¯dxdy=∑j=0K−1∑i=0N∑a=0i¯⋃i′⋃j′i¯aNiK−1jKμh2mh2μf1mf1ρi¯−a−1j+iδi¯Aj′Bj′Ai′Bi′e−μh1iδΓmh2Γmf1×∫0∞xmf1−1e−μf1x∫δ¯xϖρ+1∞ymh2+j¯+i¯−a−1e−yμh21+j+μh1iδρdxdy=∑j=0K−1∑i=0N∑a=0i¯∑s=0mh2+j¯+i¯−a−1⋃i′⋃j′i¯aNiK−1j×KΓmh2+j¯+i¯−aδ¯sμh2mh2μf1mf1ρi¯−a−1j+iδi¯Aj′Bj′Ai′Bi′e−μh1iδ−δ¯μh21+j+μh1iδρs!μh21+j+μh1iδρmh2+j¯+i¯−a−sΓmh2Γmf1×∫0∞xmf1−1e−xμf1+δ¯ϖρμh21+j+μh1iδρxϖρ+1sdx=∑j=0K−1∑i=0N∑a=0i¯∑s=0mh2+j¯+i¯−a−1∑d=0s⋃i′⋃j′sdi¯aNiK−1j×δ¯sμh2mh2μf1mf1ϖs−d−1j+iδi¯Aj′Bj′Ai′Bi′e−μh1iδ−δ¯μh21+j+μh1iδρs!μh21+j+μh1iδρmh2+j¯+i¯−a−sΓmh2Γmf1×KΓmh2+j¯+i¯−aρd+a−i¯−s∫0∞xmf1+s−d−1e−xμf1+δ¯ϖρμh21+j+μh1iδρdx=∑j=0K−1∑i=0N∑a=0i¯∑s=0mh2+j¯+i¯−a−1∑d=0s⋃i′⋃j′sdi¯aNiK−1j×KΓmh2+j¯+i¯−aΓmf1+s−dδ¯sμh2mh2μf1mf1ϖs−d−1j+is!Γmh2Γmf1μh21+j+μh1iδρmh2+j¯+i¯−a−sρd+a−i¯−s×δi¯Aj′Bj′Ai′Bi′e−μh1iδ−δ¯μh21+j+μh1iδρμf1+δ¯ϖρμh21+j+μh1iδρmf1+s−d.

For manipulation of $\varsigma_{3,1}$, we adopt (3.351.2) and (3.351.3) in [31]. Plugging (A11) and (A12) into (A10), $\varsigma_{3}$ is expressed as

(A13) ς3=∑j=0K−1⋃j′∑r=0mh2+j¯−1∑t=0rrtK−1jΓmh2+j¯r!Γmh2Γmf1×Γmf1+r−tδ¯rNμh2mh2μf1mf1−1jAj′Bj′e−δ¯μh21+jμh2mh2+j¯−r1+jmh2+j¯−rμf1+δ¯ϖρμh21+jmf1+r−t−∑j=0K−1∑i=0N∑a=0i¯∑s=0mh2+j¯+i¯−a−1∑d=0s⋃i′⋃j′sdi¯aNiK−1j×KΓmh2+j¯+i¯−aΓmf1+s−dδ¯sμh2mh2μf1mf1ϖs−ds!Γmh2Γmf1μh21+j+μh1iδρmh2+j¯+i¯−a−s×−1j+iδi¯Aj′Bj′Ai′Bi′e−μh1iδ−δ¯μh21+j+μh1iδρρd+a−i¯−sμf1+δ¯ϖρμh21+j+μh1iδρmf1+s−d.

Next, we expand $\varsigma_{4}$ defined in (A9) as

(A14) $$\begin{array}{cc} \varsigma_{4} & ≜\mathrm{Pr}\left\{{\left|g_{1,n^{*}}\right|}^{2}>\delta \chi ,{\left|g_{1,n^{*}}\right|}^{2}>\bar{\phi }\left({\left|f_{2}\right|}^{2}\varpi \rho +1\right)\right\}\\ \; & =\underset{\overset{\Delta }{\;=}\varsigma_{4,1}}{\underset{︸}{\mathrm{Pr}\left\{{\left|g_{1,n^{*}}\right|}^{2}>\delta \chi ,{\left|f_{2}\right|}^{2}<\frac{\delta \chi }{\varpi \rho \bar{\phi }}-\frac{1}{\varpi \rho }\right\}}}+\underset{\overset{\Delta }{\;=}\varsigma_{4,2}}{\underset{︸}{\mathrm{Pr}\left\{{\left|g_{1,n^{*}}\right|}^{2}>\bar{\phi }\left({\left|f_{2}\right|}^{2}\varpi \rho +1\right),{\left|f_{2}\right|}^{2}>\frac{\delta \chi }{\varpi \rho \bar{\phi }}-\frac{1}{\varpi \rho }\right\}}}, \end{array}\;$$

where $\varsigma_{4,1}$ is defined and expanded as

(A15) ς4,1≜Prg1,n*2>δχ,f22<δχϖρϕ¯−1ϖρ=∫0δχϖρϕ¯−1ϖρff22xdx1−Fg1,n*2δχdx=∑k=0N⋃k′Nk−1kAk′Bk′δk¯χk¯e−μg1kδχμ2mf2Γmf2∫0Φxmmf2−1e−μf2xdx=∑k=0N⋃k′Nk−1kAk′Bk′δk¯χk¯e−μg1kδχΓmf2γmf2,Φμf2

and

(A16) ς4,2=Prg1,n*2>ϕ¯f22ϖρ+1,f22>Φ=∫Φ∞ff22x1−Fg1,n*2ϕ¯xϖρ+1dx=∑k=0N⋃k′Nk−1kAk′Bk′μ2mf2ϕ¯k¯e−μg1kϕ¯Γmf2∫Φ∞xmmf2−1e−μf2xxϖρ+1k¯e−μg1kϕ¯ϖρxdx=∑k=0N∑c=0k¯⋃k′k¯cNk−1kϖcρcAk′Bk′μ2mf2ϕ¯k¯e−μg1kϕ¯Γmf2∫Φ∞xmmf2+c−1e−xμf2+μg1kϕ¯ϖρdx=∑k=0N∑c=0k¯⋃k′k¯cNk−1kϖcρcAk′Bk′μ2mf2ϕ¯k¯e−μg1kϕ¯Γmf2μf2+μg1kϕ¯ϖρmmf2+cΓmmf2+c,μf2+μg1kϕ¯ϖρΦ.

The last equalities of (A15) and (A16) are obtained by applying (3.381.1) and (3.381.3) in [31], respectively. By plugging (A15) and (A16) into (A14), we obtain

(A17) ς4=∑k=0N⋃k′Nk−1kAk′Bk′δk¯χk¯e−μg1kδχΓmf2γmf2,Φμf2+∑k=0N∑c=0k¯⋃k′k¯cNk−1kϖcρcAk′Bk′μ2mf2ϕ¯k¯e−μg1kϕ¯Γmf2μf2+μg1kϕ¯ϖρmmf2+cΓmmf2+c,μf2+μg1kϕ¯ϖρΦ,

where $\Phi =\frac{\delta \chi }{\varpi \rho \bar{\phi }}-\frac{1}{\varpi \rho }$, $\Gamma \left(\cdot ,\cdot \right)$ is the upper incomplete Gamma function and $\gamma \left(\cdot ,\cdot \right)$ is the lower incomplete Gamma function. By using (A13) and (A17), we can rewrite (A9) as

(A18) P1ipSIC=1−∑j=0K−1⋃j′∑r=0mh2+j¯−1∑t=0rrtK−1jΓmh2+j¯r!Γmh2Γmf1×Γmf1+r−tδ¯rNμh2mh2μf1mf1−1jAj′Bj′e−δ¯μh21+jμh2mh2+j¯−r1+jmh2+j¯−rμf1+δ¯ϖρμh21+jmf1+r−t−∑j=0M−1∑i=0N∑a=0i¯∑s=0mh2+j¯+i¯−a−1∑d=0s⋃i′⋃j′sdi¯aNiK−1j×KΓmh2+j¯+i¯−aΓmf1+s−dδ¯sμh2mh2μf1mf1ϖs−ds!Γmh2Γmf1μh21+j+μh1iδρmh2+j¯+i¯−a−s×−1j+iδi¯Aj′Bj′Ai′Bi′e−μh1iδ−δ¯μh21+j+μh1iδρρd+a−i¯−sμf1+δ¯ϖρμh21+j+μh1iδρmf1+s−d×∑k=0N⋃k′Nk−1kAk′Bk′δk¯χk¯e−μg1kδχΓmf2γmf2,Φμf2+∑k=0N∑c=0k¯⋃k′k¯cNk−1kϖcρcAk′Bk′μ2mf2ϕ¯k¯e−μg1kϕ¯Γmf2μf2+μg1kϕ¯ϖρmmf2+c×Γmmf2+c,μf2+μg1kϕ¯ϖρΦ,

which is (15).

## Appendix C. Derivation of (17)

We write (16) as

(A19) $$\begin{array}{cc} P_{1}^{pSIC}\; & =1-\underset{\overset{\Delta }{\;=}\varsigma_{5}}{\underset{︸}{\mathrm{Pr}\left\{{\left|h_{n^{*},1}\right|}^{2}>\delta \left({\left|h_{k^{*},2}\right|}^{2}\rho +1\right),{\left|h_{k^{*},2}\right|}^{2}>\bar{\delta }\right\}}}\times \underset{\overset{\Delta }{\;=}\varsigma_{6}}{\underset{︸}{\mathrm{Pr}\left\{{\left|g_{1,n^{*}}\right|}^{2}>\Lambda \right\}}}, \end{array}$$

where $\varsigma_{5}$ and $\varsigma_{6}$ are defined and expanded as

(A20) ς5=Prhn*,12>δhk*,22ρ+1,hk*,22>δ¯=∫δ¯∞fhk*,22x1−Fhn*,12δxρ+1dx=∫δ¯∞fhk*,22xdx−∫δ¯∞fhk*,22xFhn*,12δxρ+1dx=∑j=0K−1∑r=0j¯+mh2−1⋃j′K−1jK−1jδ¯rΓj¯+mh2Aj′Bj′r!Γmh2μh2j¯−rj+1j¯+mh2−re−δ¯μh2j+1−∑j=0K−1∑i=0N∑a=0i¯∑s=0j¯+mh2+i¯−a−1⋃i′⋃j′i¯aNiK−1j×Kμh2mh2−1j+iΓj¯+mh2+i¯−aδi¯δ¯sρi¯−aAj′Bj′Ai′Bi′e−μh1iδ−δ¯μh2j+1+μh1iδρs!Γmh2μh2j+1+μh1iδρj¯+mh2+i¯−a−s

and

(A21) ς6=Prg1,n*2>Λ=1−Fg1,n*2Λ=1−∑k=0N⋃k′Nk−1kAk′Bk′Λk¯e−μg1kΛ.

Plugging (A20) and (A21) into (A19), we obtain $P_{1}^{pSIC}$ as

(A22) P1pSIC=1−∑j=0K−1∑r=0j¯+mh2−1⋃j′K−1jK−1jδ¯rΓj¯+mh2Aj′Bj′r!Γmh2μh2j¯−rj+1j¯+mh2−re−δ¯μh2j+1−∑j=0K−1∑i=0N∑a=0i¯∑s=0j¯+mh2+i¯−a−1⋃i′⋃j′jaNiK−1j×Kμh2mh2−1j+iΓj¯+mh2+i¯−aδi¯δ¯sρi¯−aAj′Bj′Ai′Bi′e−μh1iδ−δ¯μh2j+1+μh1iδρs!Γmh2μh2j+1+μh1iδρj¯+mh2+i¯−a−s×1−∑k=0N⋃k′Nk−1kAk′Bk′Λk¯e−μg1kΛ,

which is result reported in (17). This is end of the proof.

## References

[1] Karagiannis G., Altintas O., Ekici E., Heijenk G., Jarupan B., Lin K., Weil T. (2011). Vehicular networking: A survey and tutorial on requirements, architectures, challenges, standards and solutions. IEEE Commun. Surv. Tutor..

[2] Yang H., Zheng K., Zhao L., Zhang K., Chatzimisios P., Teng Y. (2017). High reliability and low latency for vehicular networks: Challenges and solutions. arXiv.

[3] Sun S., Hu J., Peng Y., Pan X. (2016). Support for vehicle-to-everything services based on LTE. IEEE Wirel. Commun..

[4] Chen S., Hu J., Shi Y., Zhao L. (2018). Technologies, standards and applications of LTE-V2X for vehicular networks. Telecommun. Sci..

[5] Soni T., Ali A.R., Ganesan K., Schellmann M. Adaptive numerology—A solution to address the demanding QoS in 5G-V2X. Proceedings of the IEEE WCNC.

[6] Song L., Li Y., Ding Z., Poor H.V. (2017). Resource management in nonorthogonal multiple access networks for 5G and beyond. IEEE Netw..

[7] Saito Y., Kishiyama Y., Benjebbour A., Nakamura T., Li A., Higuchi K. Non-orthogonal multiple access (NOMA) for cellular future radio access. Proceedings of the IEEE VTC’13.

[8] Sun Q., Han S., Chin-Lin I., Pan Z. (2015). On the ergodic capacity of MIMO NOMA systems. IEEE Wirel. Commun. Lett..

[9] Zheng K., Zheng Q., Chatzimisios P., Xiang W., Zhou Y. (2015). Heterogeneous vehicular networking: A survey on architecture, challenges, solutions. IEEE Commun. Surv. Tutor..

[10] Dai L., Wang B., Yuan Y., Han S., Chih-Lin I., Wang Z. (2015). Non-orthogonal multiple access for 5G: Solutions, challenges, opportunities, future research trends. IEEE Commun. Mag..

[11] Do D.-T., van Nguyen M.-S., Hoang T.-A., Lee B.-M. (2019). Exploiting Joint Base Station Equipped Multiple Antenna and Full-Duplex D2D Users in Power Domain Division Based Multiple Access Networks. Sensors.

[12] Chen X., Liu G., Ma Z., Yu F.R., Ding Z. Power Allocation for Cooperative Non-Orthogonal Multiple Access Systems. Proceedings of the IEEE Global Communications Conference.

[13] Zhang L., Liu J., Xiao M., Wu G., Liang Y.-C., Li S. (2017). Performance Analysis and Optimization in Downlink NOMA Systems With Cooperative Full-Duplex Relaying. IEEE J. Sel. Areas Commun..

[14] Do D.-T., Le A.-T., Le C.-B., Lee B.M. (2019). On Exact Outage and Throughput Performance of Cognitive Radio based Non-Orthogonal Multiple Access Networks With and Without D2D Link. Sensors.

[15] Do D.-T., Le A.-T., Lee B.-M. (2019). On Performance Analysis of Underlay Cognitive Radio-Aware Hybrid OMA/NOMA Networks with Imperfect CSI. Electronics.

[16] Fang Z., Lu Y., Shi J., Jin L., Ji J. A Non-Orthogonal Multiple Access based Relaying Scheme for Cellular Two-Way Relay Networks. Proceedings of the International Conference on Information Science and Systems.

[17] Do D.-T., van Nguyen M.-S. (2019). Device-to-device transmission modes in NOMA network with and without Wireless Power Transfer. Comput. Commun..

[18] Do D.-T., Vaezi M., Nguyen T.-L. Wireless Powered Cooperative Relaying using NOMA with Imperfect CSI. Proceedings of the of IEEE Globecom Workshops (GC Wkshps).

[19] Nguyen T.-L., Do D.-T. (2018). Exploiting Impacts of Intercell Interference on SWIPT-assisted Non-orthogonal Multiple Access. Wirel. Commun. Mob. Comput..

[20] Do D.-T., Le A.-T. (2019). NOMA based cognitive relaying: Transceiver hardware impairments, relay selection policies and outage performance comparison. Comput. Commun..

[21] Di B., Song L., Li Y., Han Z. (2018). V2X meets NOMA: Non-orthogonal multiple access for 5G-enabled vehicular networks. IEEE Wirel. Commun..

[22] Di B., Song L., Li Y., Li G.Y. (2017). Non-orthogonal multiple access for high-reliable and low-latency V2X communications in 5G systems. IEEE J. Sel. Areas Commun..

[23] Di B., Song L., Li Y., Li G.Y. NOMA-based low-latency and high-reliable broadcast communications for 5G V2X services. Proceedings of the IEEE GLOBECOM’ 17.

[24] Khoueiry B.W., Soleymani M.R. An efficient NOMA V2X communication scheme in the internet of vehicles. Proceedings of the IEEE 85th Vehicular Technology Conference (VTC Spring).

[25] Chen Y., Wang L., Ai Y., Jiao B., Hanzo L. (2017). Performance analysis of NOMA-SM in vehicle-to-vehicle massive MIMO channels. IEEE J. Sel. Areas Commun..

[26] Liu G., Wang Z., Hu J., Ding Z., Fan P. (2019). Cooperative NOMA Broadcasting/Multicasting for Low Latency and High-Reliability 5G Cellular V2X Communications. IEEE Internet Things J..

[27] Guo S., Zhou X. Robust power allocation for NOMA in heterogeneous vehicular communications with imperfect channel estimation. Proceedings of the IEEE Annu. Int. Symp. PIMRC.

[28] Qian L.P., Wu Y., Zhou H., Shen X. (2017). Dynamic cell association for non-orthogonal multiple-access V2S networks. IEEE J. Sel. Areas Commun..

[29] Yue X., Liu Y., Kang S., Nallanathan A., Chen Y. (2018). Modeling and analysis of two-way relay non-orthogonal multiple access systems. IEEE Trans. Commun..

[30] Wang X., Jia M., Ho I.W., Guo Q., Lau F.C.M. (2019). Exploiting full-Duplex Two-way relay cooperative Non-Orthogonal multiple access. IEEE Trans. Commun..

[31] Gradshteyn I.S., Ryzhik I.M. (2000). Table of Integrals, Series and Products.

